# The Impact of Match Location and Players’ Physical and Technical Activities on Winning in the German Bundesliga

**DOI:** 10.3389/fpsyg.2020.01748

**Published:** 2020-07-23

**Authors:** Marek Konefał, Paweł Chmura, Antonio Tessitore, Tomasz Melcer, Edward Kowalczuk, Jan Chmura, Marcin Andrzejewski

**Affiliations:** ^1^Department of Biological and Motor Sport Bases, University School of Physical Education in Wrocław, Wrocław, Poland; ^2^Department of Team Sport Games, University School of Physical Education in Wrocław, Wrocław, Poland; ^3^Department of Movement, Human and Health Sciences, University of Rome “Foro Italico”, Rome, Italy; ^4^Department of Biomedical Engineering, Faculty of Fundamental Problems of Technology, Wrocław University of Science and Technology, Wrocław, Poland; ^5^Football Club Hannover 96, Hanover, Germany; ^6^Department of Methodology of Recreation, Poznań University School of Physical Education, Poznań, Poland

**Keywords:** match analysis, elite football, high intensity, seasons, close matches

## Abstract

This study aimed to examine whether the physical and technical activities of soccer players and match locations can be associated with higher or lower odds of winning matches whose outcome can be described as “close.” The study comprised 7972 individual observations of German Bundesliga players during the 2014/2015 (*n* = 2794), 2015/2016 (*n* = 2494), and 2016/2017 (*n* = 2684) seasons. A selection of “close matches” was made, which were defined as those in which the difference in numbers of scored goals was ≤1. Players’ five pitch positions were considered: central defenders, fullbacks, central midfielders, wide midfielders, and forwards. Data on 12 physical and 10 technical activities performed by players during matches as well as on match location were retrieved from the Impire AG (Germany) match analysis system. The study results show that the odds of winning at home are different for each playing position: from 41.99% for wide midfielders to 91.34% for central midfielders. Another conclusion was that one of the key components of the predictive model for forwards is the percentage of overall distance covered at speeds >24 km/h (7.99%), which is a variable with an increasing trend. The proposed model predicts that each 1% increase in this variable will theoretically be associated with a 4.08% raise of the odds of winning in further seasons. The presented statistical model may be used by trainers to identify players’ physical and technical activities and contextual variables that may significantly affect the match outcome. In addition, it can help to determine the individual training load related to the player’s position on the pitch.

## Introduction

Notational analysis plays a very important role in all sport games, particularly in soccer (Carling et al., 2009; Sarmento et al., 2014). Consequently, match analysis studies can provide useful information for coaches, who need significant, relevant, and easily gathered feedback to be successfully applied in training and competitive matches (Hughes and Franks, 2004; Wright et al., 2013). Today, technologically advanced systems are commonly used for data gathering (Castellano et al., 2014) based on the state-of-the-art algorithms along with video-recording technology to supply data on the physical (motion analysis) and technical and tactical (notational analysis) performance of soccer players (Bradley et al., 2013; Konefał et al., 2019a) with regard to their playing positions (Andrzejewski et al., 2018) and contextual variables (Chmura et al., 2017b; Gai et al., 2019; Oliva-Lozano et al., 2020).

During a soccer match, players are engaged in a number of multidirectional physical and technical activities (Krustrup et al., 2005; Wallace and Norton, 2014); however, relevant research on soccer shows that players’ technical activities can be better predictors of success than their physical activities (Castellano et al., 2012; Carling, 2013; Di Salvo et al., 2013; Hoppe et al., 2015; Moalla et al., 2017). Among the many technical variables used in soccer match analysis, the most important are considered to be shots, passes, and one-on-one plays (Lago-Penas and Lago-Ballesteros, 2011; Castellano et al., 2012; Liu et al., 2015, 2016; Szwarc et al., 2017). Their high effectiveness is correlated with the match outcome (Shafizadeh et al., 2013; Link and de Lorenzo, 2016; Konefał et al., 2018). The achievement of players’ high tactical and technical levels is not possible without players’ appropriate levels of physical activity (Mackenzie and Cushion, 2013), whose main measurements include total distance covered, distance covered at high intensity, number of performed sprints, mean running speed, and peak running speed (Andrzejewski et al., 2018; Chmura et al., 2018). A study of Bundesliga players revealed that the highest odds of winning a match (31.7%) occur when wide midfielders increase their distance covered at above 24 km/h by 0.1 km. Likewise, for fullbacks, increasing the number of sprints will improve the odds of winning a match by 8.6% (Konefał et al., 2019a).

However, even if the multiple physical and technical variables are regarded as individual aspects of a match, a reductionist approach to them and their separate analyses cannot offer a full reflection of the complexity of all characteristics of a soccer game (Mackenzie and Cushion, 2013). On the one hand, when more detailed consideration is given to the various playing positions, it can be noted that, during the match, the central midfielders perform a higher number of passes and are characterized by the highest passing accuracy, and the forwards perform the highest numbers of shots and shots on target (Bradley et al., 2013). Likewise, the effectiveness of a game played by fullbacks and wide midfielders is associated with the distance covered at high intensity as well as with the number of performed sprints (Andrzejewski et al., 2018; Chmura et al., 2018). In essence, this means that improving the team’s success requires a higher level of physical activity from players on some pitch positions and enhanced technical activity from players in others (Di Salvo et al., 2013). On the other hand, these analyses should not be separated from the contextual variables, such as match outcome (Rumpf et al., 2017) and match location, which are also highly correlated with each other (Lago-Penas et al., 2016). Pollard and Pollard (2005) analyzed more than 400,000 matches over the period of 1876–2003 to identify a home-advantage trend in soccer and other team sports games. Therefore, the observed changes in players’ activities in subsequent seasons are an important factor (Barnes et al., 2014). An interesting analysis of the World Cup Finals between 1966 and 2010 showed that every single variable considered changed significantly over time (Wallace and Norton, 2014). Bush et al. (2015) also demonstrated that the overall numbers of passes increased by 40% over seven consecutive seasons of the English Premier League.

In addition, researchers indicate a number of other determinants of players’ activity during a match, including quality of opposition (Bilek and Ulas, 2019), team and opposition ability (Redwood-Brown et al., 2019), team formation (Aquino et al., 2020), match status (Gonzalez Rodenas et al., 2019), players’ age and experience (Lorenzo-Martínez et al., 2019), and climate conditions (Coker et al., 2020). However, during a soccer match, players experience all these factors simultaneously, which is why modern studies focus on multifactorial analyses, using advanced statistical methods (Moura et al., 2014). Taylor et al. (2008) used logistic regression to develop a model to assess the frequency of on-the-ball technical activities performed by a professional British soccer team as a function of match location and quality of opposition in relation to match outcome. Aquino et al. (2020) noted that matches played at home or against “weaker” opponents presented greater running demands and that match outcome demonstrated influence only for running performance; matches in which the reference team won resulted in higher values than in matches lost. Bilek and Ulas (2019) used the decision tree identifying scoring first as the most influential on match outcome in each decision tree, and the impact of match location on the match outcome varied according to the quality of the opponent. Multivariate analyses (adjusted logistic multilevel models) by Gonzalez Rodenas et al. (2019) showed how playing at home and against high-ranked opponents involved a higher likelihood of achieving offensive penetration although no associations were found in terms of creation of scoring opportunities. There is a significant relationship between match location, quality of opposition, and other contextual variables affecting the physical and technical activities of soccer players.

An interesting point of further exploration of this issue may be the combination into one multivariate analysis of match locations with players’ pitch positions and the match outcome. We know that each playing position displays different physical and technical activities, and this depends on the match outcome (Sarmento et al., 2014). It can be assumed that such differences in physical and technical activity will also occur when such an important contextual factor as match location is considered in the statistical model. It may be particularly interesting to find out how the home or away games affect players in different pitch positions. In addition, researchers have demonstrated that a specific winning and losing margin should be employed to identify the closeness of a game score and that only close matches can be seen as representative of the best performance between two opposing teams (Gomez et al., 2014; Liu et al., 2016).

Considering the abovementioned information, the present study aimed to examine whether different levels of players’ physical and technical activities and match location were associated with higher or lower odds of winning or losing close matches of the German Bundesliga.

## Materials and Methods

### Participants

The study material consisted of observations (*n* = 556) from Bundesliga soccer players playing in three seasons from 2014/2015 to 2016/2017. Of all studied players, 228 played one season, 167 played two seasons, and 161 played three seasons. The number of studied players in subsequent seasons was as follows: 2014/2015 (*n* = 349), 2015/2016 (*n* = 344), and 2016/2017 (*n* = 352). The study material comprised 13,032 individual observations of activities of outfield players (goalkeepers were excluded). Only players who played whole matches were selected for the study.

In total, 918 matches were assigned to one of two categories: (i) close matches (7972 match observations) during 2014/2015 (*n* = 2794), 2015/2016 (*n* = 2494), and 2016/2017 (*n* = 2684) in which the difference in match score was up to 1 goal (≤1) and (ii) unbalanced games (5060 match observations) in which the difference in match score was two or more goals (≥2) (Liu et al., 2016). Further analyses were confined to close matches. Moreover, five pitch positions of players were considered (Bradley et al., 2013; Andrzejewski et al., 2018; Chmura et al., 2018): central defenders (CD, 2210 match observations), fullbacks (FB, 1691 match observations), central midfielders (CM, 1963 match observations), wide midfielders (WM, 1159 match observations), and forwards (F, 949 match observations).

The study conformed with the Declaration of Helsinki and was approved by the local Ethics Committee (No. 339/15). The study protocol was also approved by the local board of ethics.

### Procedures

The notational analysis of 918 played matches was carried with the use of the Impire AG match analysis system (Germany) (Tiedemann et al., 2011) with a recording frequency of 25 Hz. Each player’s movement was recorded by two cameras (Link and Weber, 2017). The system utilizes state-of-the-art algorithms and 2-D and 3-D video-recording technology, allowing for detailed motion analysis of entire soccer matches. The major advantages of vision-based systems are their high update rate corresponding to the camera-frame rate and the fact that the players and the ball are tracked simultaneously. The validity and reliability of this system have been described in detail elsewhere (Liu et al., 2013).

Performance-related activities were divided into two groups: (1) physical variables: total distance [km] covered during the match; distance [km] covered at speeds below or above 14.4 km⋅h^–1^ (below or above the anaerobic threshold); distance [km] and the percentage of distance covered at intensity ranges <11 km⋅h^–1^, 11–14 km⋅h^–1^, 14–17 km⋅h^–1^, 17–21 km⋅h^–1^, 21–24 km⋅h^–1^, and >24 km⋅h^–1^; numbers of performed sprints during a match (runs with a speed >22.68 km⋅h^–1^ and at least 1 s in duration); numbers of efforts at high intensity (above 18 km⋅h^–1^); mean running speed [km⋅h^–1^]; and peak running speed [km⋅h^–1^] (Andrzejewski et al., 2018; Chmura et al., 2018) and (2) technical variables: numbers of shots, i.e., attempts to score a goal made with any (legal) part of the body, either on or off target; numbers of ball touches; numbers of passes, i.e., intentionally played balls from one player to another; pass accuracy [%], i.e., successful passes as a proportion of total passes; numbers of crosses, i.e., any balls sent into the opposition team’s area from a wide position; numbers of one-on-one plays (game actions in which two players from different teams compete for the ball, and the action is always assigned to both participant players); and number and percentage of one-on-one plays won (Konefał et al., 2019a). Additionally, the study also considered the contextual variable of match location, i.e., whether a team was playing at its own ground (1 = Home) or at its opponent’s (0 = Away) (Gonzalez Rodenas et al., 2019). Complete definitions of physical and technical variables are available at the DFL | Definitionskatalog Offizielle Spieldaten – Bundesliga website^1^.

For each variable of physical or technical activity performed by a player in a given match, an extra variable was set measuring the change in the coefficient in three consecutive seasons. These additional variables were defined as an interaction term between the original variable and the season’s sequential number, i.e., 0 for the 2014/2015 season, 1 for the 2015/2016 season, and 2 for the 2016/2017 season (Tibshirani, 2011).

### Statistical Analysis

Statistical analysis was carried out using the STATISTICA ver. 13.1 (StatSoft Inc., United States) software package and the *glmnet* R package for regression analysis (Tibshirani, 2011). The selection of physical and technical activities for the predictive models was made using the lasso method (Tibshirani, 2011), i.e., a method for variable selection by regularization, applicable to a large class of linear models. The lasso penalizes models by adding an L^1^ norm of the model parameters to the cost function. The L^1^ norm favors models with many coefficients set to zero, effectively performing feature selection by suppressing variables that have a small influence on the target variable, weak predictors correlated to stronger variables, and, in this particular case, variables for which it was not possible to decide on the direction of the effect on match outcome on the basis of the analyzed data set (Konefał et al., 2019a). The lasso method is preferred to the practice of choosing variables by reference to *p*-values of statistical tests or methods, such as forward feature selection or backward feature elimination, due to the fact that variable selection is an inherent part of model construction, and hence, parameter estimates as well as various model test statistics are more reliable (Tibshirani, 2011; Harrell, 2015). Logistic regression with the lasso penalty was applied with the match outcome as the dichotomous variable: 1 = win, 0 = loss or draw. The model was estimated using the least-angle regression method (Efron et al., 2014) with the permutation test for model components also being applied as suggested by Tibshirani (2011). The level of statistical significance for the permutation test was set at α = 0.05. It should be noted here that the use of the chi-square or Wald tests would result in biased confidence interval estimates when applied directly to the results obtained using the lasso method (Tibshirani, 2011).

## Results

The descriptive statistics for the examined variables concerning players’ pitch positions in close matches are shown in Table 1.

**TABLE 1:** Physical and technical activities of players in different pitch positions in close games (means and SD).

Variables	Close games (≤1)				
	F	WM	CM	FB	CD
Total distance covered [km]	10.70 ± 0.90	11.19 ± 0.71*	11.55 ± 0.70*	10.72 ± 0.60	10.03 ± 0.61
Distance covered below 11 km/h [km]	6.29 ± 0.30*	6.28 ± 0.31	6.20 ± 0.30*	6.24 ± 0.32*	6.36 ± 0.31
Distance covered at 11–14 km/h [%]	15.65 ± 2.30	16.47 ± 1.89	19.07 ± 2.10*	16.57 ± 1.63	16.49 ± 1.99
Distance covered below 14.4 km/h [km]	8.16 ± 0.50*	8.33 ± 0.43	8.66 ± 0.40	8.21 ± 0.41	8.19 ± 0.42
Distance covered at 14–17 km/h [%]	10.25 ± 1.8	11.20 ± 1.59	13.19 ± 2.10*	10.76 ± 1.45	9.58 ± 1.59
Distance covered at speeds >24 km/h [km]	0.33 ± 0.10	0.37 ± 0.13*	0.21 ± 0.10	0.31 ± 0.11	0.16 ± 0.08
Distance covered at speeds >24 km/h [%]	3.06 ± 1.20*	3.30 ± 1.15	1.80 ± 0.90	2.87 ± 1.01	1.61 ± 0.74
Distance covered at speeds >24 km/h – seasons [%]	3.06 ± 1.20*	3.30 ± 1.15	1.80 ± 0.90	2.87 ± 1.01	1.61 ± 0.74
Sprints [number]	24.44 ± 7.70*	26.36 ± 7.40	17.00 ± 6.60*	22.33 ± 6.37	12.97 ± 4.88
Sprints – seasons [number]	24.44 ± 7.70	26.36 ± 7.40	17.00 ± 6.60*	22.33 ± 6.37	12.97 ± 4.88
Efforts at high intensity above 18 km/h [number]	42.47 ± 10.90	45.45 ± 9.96	47.48 ± 12.30*	40.03 ± 8.59	30.33 ± 7.64
Peak running speed [km/h]	31.49 ± 1.60*	31.82 ± 1.41	30.20 ± 1.60*	31.46 ± 1.31	30.58 ± 1.64
Mean running speed [km/h]	6.81 ± 0.50	7.11 ± 0.47	7.34 ± 0.50*	6.82 ± 0.40	6.38 ± 0.40
Total shots [number]	2.54 ± 1.69*	1.81 ± 1.55	1.15 ± 1.27*	0.54 ± 0.81	0.58 ± 0.83
Total shots – seasons [number]	2.54 ± 1.69*	1.81 ± 1.55	1.15 ± 1.27	0.54 ± 0.81	0.58 ± 0.83
Passes [number]	23.39 ± 9.79	32.17 ± 12.77*	47.07 ± 20.38*	39.26 ± 15.59	49.18 ± 24.53
Pass accuracy [%]	66.76 ± 12.43*	69.99 ± 11.91	76.45 ± 10.92	71.29 ± 12.13	78.55 ± 11.88
Crosses [number]	0.89 ± 1.44	2.16 ± 2.22*	0.69 ± 1.33*	1.92 ± 1.85*	0.10 ± 0.49
Crosses – seasons [number]	0.89 ± 1.44*	2.16 ± 2.22	0.69 ± 1.33	1.92 ± 1.85	0.10 ± 0.49
One-on-one plays [number]	25.10 ± 8.84*	22.71 ± 7.85	22.17 ± 7.40	17.95 ± 6.09	15.23 ± 5.55
One-on-one plays won [number]	10.99 ± 4.61	10.72 ± 4.48	11.23 ± 4.52*	9.96 ± 4.02	9.19 ± 3.97
One-on-one plays won – seasons [number]	10.99 ± 4.61	10.72 ± 4.48	11.23 ± 4.52*	9.96 ± 4.02	9.19 ± 3.97
Percentage of one-on-one plays won	43.92 ± 10.78	47.37 ± 11.91	50.66 ± 12.06*	55.42 ± 12.62	60.17 ± 14.46
Playing at home	0.001*	0.001*	0.001*	0.001*	0.001*

*Variables selected for the model with the use of the lasso method due to their predictive power. Pitch positions: forwards (F), wide midfielders (WM), central midfielders (CM), fullbacks (FB), and central defenders (CD).

In close matches, as regards to forwards, the statistically significant model (chi^2^ *p*-value < 0.040) revealed statistical significance in relation to distance covered at speeds below 14.4 km⋅h^–1^ (*p* < 0.001), percentage distance covered at speeds >24 km⋅h^–1^ (*p* < 0.026), percentage distance covered at speeds >24 km⋅h^–1^ – seasons (*p* < 0.004), number of sprints (*p* < 0.014), total number of shots (*p* < 0.001), total number of shots – seasons (*p* < 0.012), the number of one-on-one plays (*p* < 0.044), and playing at home (*p* < 0.001) (Table 2).

**TABLE 2:** Relationships between selected variables and match outcomes among elite German soccer players.

Parameters	Close games (≤1)				
	F	WM	CM	FB	CD
Total distance covered (increase by 0.1 km)		**55.94**	+*		
Distance covered below 11 km/h (increase by 0.1 km)			+*	+*	
Distance covered below 14.4 km/h (increase by 0.1 km)	+*				
Distance covered at 14–17 km/h (increase by 1 %)			**8.81**		
Distance covered at speeds >24 km/h (increase by 0.1 km)		**60.71**			
Distance covered at speeds >24 km/h (increase by 1 %)	**7.99**				
Distance covered at speeds >24 km/h - seasons (increase by 1%)	**4.08**				
Number of sprints (unit increase)	**1.41**				
Mean running speed (increase by 0.1 km/h)			–*		
Total number of shots (unit increase)	**11.44**				
Total number of shots - seasons (unit increase)	**2.72**				
Number of passes (unit increase)		**0.41**	**–0.41**		
Number of crosses (unit increase)		**–0.54**	**–9.16**	**–0.28**	
Number of one-on-one plays (unit increase)	**0.88**				
Playing at home	**51.75**	**41.99**	**91.34**	**49.34**	**59.93**

*Results marked with an asterisk (“^∗^”) were identified by visual inspection as unreliable in terms of values for the coefficient. These results have a very large confidence interval. This means that even though the model selected the variable as a contributor to the match outcome, more-precise estimate cannot be attained, but it can merely point out the direction of the variable’s effect as helpful (positive) or harmful (negative) for the match outcome.*

As far as the wide midfielders were concerned (chi^2^ *p*-value < 0.001), the statistically significant variables were total distance covered (*p* < 0.012), distance covered at speeds >24 km⋅h^–1^ (*p* < 0.031), numbers of passes (*p* < 0.005), numbers of crosses (*p* < 0.025), and playing at home (*p* < 0.001). In line with the estimated model (chi^2^ *p*-value < 0.001), the statistically significant variables for central midfielders were, in turn, total distance covered (*p* < 0.001), distance covered at speeds below 11 km⋅h^–1^ (*p* < 0.001), percentage distance covered at 14–17 km⋅h^–1^ (*p* < 0.001), mean running speed (*p* < 0.001), numbers of passes (*p* < 0.040), numbers of crosses (*p* < 0.003), and playing at home (*p* < 0.001). For fullbacks (chi^2^ *p*-value < 0.001), the statistically significant variables were distances covered at speeds below 11 km.h^–1^ (*p* < 0.007), numbers of crosses (*p* < 0.027), and playing at home (*p* < 0.001). In the estimated model (chi^2^ *p-*value < 0.001) only playing at home (*p* < 0.001) was statistically significant for central defenders (Table 2).

## Discussion

This study aimed to examine whether different levels of soccer players’ physical and technical activities and match location were associated with higher or lower odds of winning or losing close matches in the German Bundesliga. Our main findings show that match location is a contextual variable present in the estimated models for all pitch positions, having a positive effect on the odds of a close match being won. The importance of playing at home in soccer has been emphasized in many previous studies (Lago-Penas et al., 2010; Lago-Penas and Lago-Ballesteros, 2011; Liu et al., 2016), and although the significance of home advantage has declined steadily from around 70% in 1890 to around 60% today, it remains clear that match location continues to exert a major influence on the match outcome (Pollard and Pollard, 2005). The mean value of playing at home, irrespective of the five playing positions analyzed in our study, was 59%. It is a similar percentage to that observed by Pollard and Gómez (2014), who drew on data from the Spanish Professional League to determine a mean value for home advantage equal to 62%. However, our study is the first to demonstrate in a more detailed way how the odds of winning at home are different for each playing position with the range from 42% for wide midfielders to 91% for central midfielders. Furthermore, the 60% noted for central defenders represented the only component of the model relating to this position. It can be argued that central midfielders, due to the position-specific technical and tactical requirements for directing the match, are more affected by the game at home than wide midfielders and defenders. This may be due to the fact that central midfielders are characterized by longer ball possession and higher numbers of attacking behaviors (e.g., shots, dribbles, and ball touches) when playing at home (Konefał et al., 2019b). This result clearly shows that match location should be taken into account by coaching staffs in their analyses for the entire team (Liu et al., 2016) as well as individual players in different pitch positions.

However, despite the high home advantage noted in our study, the impact of match location itself should be assessed with caution. If the opponent is presenting a lower level of quality, a home match does not affect the odds of winning, but it increases the probability of a draw and reduces the probability of a loss. However, if the opponent is stronger, playing at home provides an extra 14% chance of winning (Bilek and Ulas, 2019). Other respondents indicate that playing at home and playing against high-ranked opponents increases the effectiveness of the offense but does not affect the goal-scoring opportunities (Gonzalez Rodenas et al., 2019). Other studies, however, do not confirm this “finding” (Almeida et al., 2014) and show an increase in offensive rates. It follows that even multivariate analyses may not capture the high level of complexity (e.g., Almeida et al., 2014; Gonzalez Rodenas et al., 2019) that soccer styles represent, where constant interaction between team members, opponents, and context constraints creates dynamic and interdependent situations that are different and unique in each match. A very interesting example of these complex relationships is the study showing the decision tree for games in which the quality of the team is inferior to the opponent. In such a match, without taking into account any variable, it can be concluded that the team is likely to lose the match with a probability of 0.59. However, if the team scores the first goal, it will roughly triple its chance to win. Moreover, if the team is the host, its probability of winning increases to 0.62. In addition, if the team manages to keep the percentage of possession of the ball above 44%, it is very likely that the team will win the game with a probability of 0.89 (Bilek and Ulas, 2019).

The present study shows that, as far as forwards are concerned, the odds of winning are higher in matches in which a player covers a greater percentage of distance at speeds >24 km⋅h^–1^ and performs a higher number of sprints. These results are in line with the findings by Andrzejewski et al. (2018) and Chmura et al. (2018), who showed that the fact that the forwards were able to perform more sprints and runs of high intensity was crucial to the match outcome. Moreover, Andrzejewski et al. (2018) also stressed that, in soccer, high-intensity activities occur more often in offensive play. The findings of the present study indicate that the predictive model for forwards stressed not only the greater percentage of the distance covered at speeds higher than 24 km⋅h^−1^ and also its significant improvement over the seasons. The model predicts that a value for 1% higher will be associated with a steady (4.08%) increase in the odds of winning in subsequent seasons. Previous studies also revealed a 30–50% increase in the distances covered at high intensity or by sprinting by players of the English Premier League in the last decade. However, our research emphasizes the increase in forwards’ play intensity as an ongoing process, which is essential for the improvement of the odds of winning (Bradley et al., 2013). In fact, this ability has a particular relevance for forwards because most goals are scored in the wake of dynamic actions taken by these players (Njororai, 2013). Furthermore, shots represent, in turn, a further key variable to be taken into account in developing a model for forwards. In this regard, our findings show that when a forward takes single shots more frequently during a match, the odds of winning the game increase by 11%. In turn, in further seasons, an increased chance of winning was associated with an increased frequency of this technical activity (in subsequent seasons, one extra shot increased the odds of winning by 2.72%). The importance of shots is confirmed by many studies showing that differences between match status (winning or losing) in the case of professional teams are mainly attributed to the numbers of shots at goal as well as the effectiveness thereof with the task of performing shots mainly assigned to forwards (Lago-Penas et al., 2010; Castellano et al., 2012; Liu et al., 2015). On the other hand, Barnes et al. (2014) did not observe any changes in the numbers of shots taken over seven seasons of the English Premier League. However, the present study was not confined only to forwards. In our sample, the forwards took 2.54 ± 1.69 shots on average in each match. For comparison, during the Euro 2012 championship, the mean number of shots for all matches and for all pitch positions was 16.83 ± 7.60 (Shafizadeh et al., 2013). Also during the Euro 2016 championship, one shot was taken every 7 min on average (Konefał et al., 2018). This could be explained by the fact that, in games characterized by smaller differences in the numbers of goals, far greater significance is assigned to numbers of shots taken by forwards. Moreover, shots are part of a model that makes associations between one whole entity and other variables of physical activity (distance covered at speeds greater than 24 km⋅h^–1^, number of sprints), or tactical activity (one-on-one plays) (Liu et al., 2015; Szwarc et al., 2017).

The estimated models for wide midfielders highlighted total distance covered (55.94%), distance covered at a speed >24 km⋅h^–1^ (60.71%), and number of passes (0.41%) as relevant variables that could positively influence the match outcome. In present-day soccer, wide midfielders are players who, during a match, above all others, very often cover the longest distance at high intensity (Lago-Penas et al., 2016). Interestingly, a predictive model developed by Konefał et al. (2019a) using data from the 2014/2015 Bundesliga season also indicated the importance of wide midfielders’ distance covered at speeds >24 km⋅h^–1^, albeit with the odds of winning amounting to 31.7% and the number of passes to 3.3%.

The present study is confined to the analysis of close matches and not all matches. This can account for the noted lesser significance of technical activities in the case of wide midfielders and an associated greater relevance of their physical activities manifested by their coverage of long distances as well as distances at very high intensity during a match. Nowadays, in soccer close matches, wide midfielders in possession of the ball often take part in dynamic offensive actions while following losses of the ball by covering long distances and returning to defensive tasks (Goncalves et al., 2017; Baptista et al., 2018). This is also an indication for soccer clubs recruiting wide midfielders to select those characterized by more “explosive” abilities and a capacity to engage in frequently repeated high-intensity efforts (Bush et al., 2015; Chmura et al., 2018).

As far as central midfielders are concerned, the variables related to the increase of odds of match winning included total distance covered and distance covered at speeds below 11 km⋅h^–1^. The importance of these variables was emphasized by Chmura et al. (2017a), who showed that the 2014 World Cup winning team covered a significantly greater total distance per match compared to the other teams. However, despite a positive relationship between these variables and match outcome, the model does not supply precise information on the rate of change. This may be a reflection of the major input of other external factors, such as differences in playing and coaching styles.

As regards central midfielders, the model considered the percentage of distance covered at 14–17 km⋅h^–1^, whose increase was associated with an 8.81% change in the odds of winning as well as with the negative influence of mean running speed. It may be that central midfielders’ movement on the field at such an intensity favors closer observation and perception of the situation on the pitch, thus ensuring a more effective implementation of match tasks (Chmura and Nazar, 2010). The analysis of close matches in the present study shows that the transfer of the ball to wide midfielders and forwards increases the dynamics of the game and leads to a greater number of shots being taken. However, it is also important to note that the impact of numbers of passes and crosses by central midfielders on the match outcome is negative. Bush et al. (2015) found that over the English Premier League 2006/7–2012/13 seasons central midfielders were able to achieve a 50% increase in the number of performed passes. In turn, Bradley et al. (2013) showed that it was central midfielders who were responsible for most passes. The different results in our study regarding the activity of central midfielders as well as fullbacks may point to differences in playing styles in particular national soccer leagues or to a new trend of changes in the style of play among modern soccer teams. The latter may be based on the idea that, on average, it passes from the midfield to the attacking area that proves most effective (Rein et al., 2017) and that the ratio of goals from shots is better for “direct play” than for “possession play” (Hughes and Franks, 2005). This way of soccer play could be observed in successive matches of the 2018 World Cup in Russia.

A limitation of the present study is that the statistical model should include more contextual-related variables influencing match running performance and behaviors of professional players, such as level of opposition (top, middle, bottom) and match status (win, draw, lose). Also styles of play were not considered in the study, and thus, no associations with winning or KPIs could have been examined. In addition, the present study focuses on one specific league (the Bundesliga), and therefore, the data obtained should be considered with some caution. Also adding more numbers of seasons to the database and *k*-means clustering will allow for more accurate modeling and predictions of what actions should be performed by soccer players to raise the odds of winning in further seasons. In further studies, the data should be normalized by each individual player’s contribution to his team during each match. Last, future research should also consider environmental conditions, team tactics, and players’ age/experience.

## Conclusion

An important new finding of the present study is that playing at home is the most significant determinant of match outcome for Bundesliga professional soccer players in all pitch positions. However, the odds of winning at home were found to vary with pitch position, being the lowest among wide midfielders and the highest among central midfielders. The results of close matches over three Bundesliga seasons were determined to a greater extent by the activity of offensive players than defensive players. Statistical models predicting the odds of winning a match revealed the statistical significance of distance covered at very high intensity, number of sprints, and total distance covered as well as the total numbers of shots and numbers of one-on-one plays for forwards and wide midfielders. The application of an additional variable to measure the change of coefficient for all studied variables in consecutive seasons pointed to the great significance of the evolution of the game, percentage distance covered at speeds greater than 24 km⋅h^–1^, and total number of shots among the forwards.

In comparison with analyses of all matches, the present study, which was confined to close matches only, revealed a declining significance of technical activities and increasing significance of physical activities among wide midfielders. This is manifested by players covering longer distances during matches as well as longer distances at higher intensities.

## Practical Application

The results of the present study provide practical information that can be effectively used in physical, tactical, and technical training. First, the study shows that match location is a contextual variable present in the estimated models for all pitch positions, having a positive effect on the odds of winning a close match. Second, the results of our study show that, to increase the odds of match winning, the soccer training for forwards and wide midfielders should focus more on high- and very high-intensity efforts. The offensive players must demonstrate a high level of aerobic capacity, tolerance to increasing fatigue, and anaerobic capacity. Having such potential can prevent the decrease in the players’ ability to repeat sprints. This can have a positive impact on the efficiency of technical activities, especially more shots on goal as this variable will play an increasingly important role with each subsequent season. Therefore, the above recommendations should be considered when selecting offensive players.

The presented statistical model may be used by coaching staffs to identify players’ physical and technical activities and contextual variables that may significantly affect the match outcome. In addition, it can help to determine individual training loads related to the player’s position on the pitch.

## Data Availability Statement

The raw data supporting the conclusions of this article will be made available by the authors, without undue reservation, to any qualified researcher.

## Ethics Statement

The studies involving human participants were reviewed and approved by the Local Board of Ethics. This study maintains the anonymity of the players following data protection laws, and was conducted in compliance with the Declaration of Helsinki. The patients/participants provided their written informed consent to participate in this study.

## Author Contributions

MK, PC, JC, and MA conceptualized the study and contributed to the study methodology and writing (original draft). MK, PC, and EK undertook the data curation and investigation. MK and TM were responsible for the formal analysis and visualization and contributed to the software. JC contributed to the funding acquisition. MK, AT, and JC undertook the project administration and responsible for the supervision. MK, EK, and JC contributed to the resources. MK, PC, AT, JC, and MA contributed to the validation and writing (review and editing). All authors contributed to the article and approved the submitted version.

## Conflict of Interest

The authors declare that the research was conducted in the absence of any commercial or financial relationships that could be construed as a potential conflict of interest.

## Footnotes

1 https://s.bundesliga.com/assets/doc/10000/2189_original.pdf
